# Ameliorating the Mechanical Parameters, Thermal Stability, and Wettability of Acrylic Polymer by Cement Filling for High-Efficiency Waterproofing

**DOI:** 10.3390/polym14214671

**Published:** 2022-11-02

**Authors:** Alaa M. Abd-Elnaiem, Seenaa I. Hussein, Nadia A. Ali, Ahmad Hakamy, Abdelazim M. Mebed

**Affiliations:** 1 Physics Department, Faculty of Science, Assiut University, Assiut 71516, Egypt; 2 Department of Physics, College of Science, University of Baghdad, Baghdad 10071, Iraq; 3 Department of Physics, Umm Al-Qura University, Makkah 24382, Saudi Arabia; 4 Department of Physics, College of Science, Jouf University, Sakaka 2014, Saudi Arabia

**Keywords:** acrylic polymer, cement, waterproof, mechanical, thermal stability

## Abstract

Acrylic polymer/cement nanocomposites in dark and light colors have been developed for coating floors and swimming pools. This work aims to emphasize the effect of cement filling on the mechanical parameters, thermal stability, and wettability of acrylic polymer. The preparation was carried out using the casting method from acrylic polymer coating solution, which was added to cement nanoparticles (65 nm) with weight concentrations of (0, 1, 2, 4, and 8 wt%) to achieve high-quality specifications and good adhesion. Maximum impact strength and Hardness shore A were observed at cement ratios of 2 wt% and 4 wt%, respectively. Changing the filling ratio has a significant effect on the strain of the nanocomposites. The contact angle was increased as the concentration of additives and cement increased, indicating that the synthesized coating is not hydrophilic and does not allow water permeability through it. The results show that the acrylic polymer/cement with a cement ratio of 8 wt% is the best nanocomposite for high-efficiency waterproofing.

## 1. Introduction

Surface coating with materials should be consistent with the coated surface to avoid the difficulties, such as failures, after application, or after a long time [1]. Meanwhile, the resistance to exterior weathering, water chemical resistance, abrasion, heat resistance, time resistance, and ease of application, may happen in a more stable coating [1]. Cement-based materials act as a hydraulic binder, increasing the binding between fractured particles and allowing them to be used in a variety of industries [2]. It is generally known that a composite is made up of at least two materials from the categories of metals, ceramics, and/or polymers. The goal of composite synthesis is to perform a mixture of characteristics that are not present in the separate elements and compounds [3,4]. There are several composite material processing methods and significant variances in the various types of composite material molding procedures. The molding technique is often known as the hand lay-up method. The forming procedures of composite materials and products are the compounding of particles and polymers and the curing reaction process of the resin system [5,6].

Thin-film coatings of organic composites are composed of an organic polymeric binder and other elements, such as pigments, fillers, solvents, water, organic cross-linkers, etc. The organic adhesive is the continuous phase that connects the filler and pigment, and the latter is used to provide color, protect against corrosion, and alter the barrier qualities. Most of the fillers are expensive and to consume the costs, calcium carbonate-based materials such as cement can be used to substitute parts of the more costly coating components [7,8].

Wettability is a term used to describe a liquid’s ability to spread across a solid surface, and the contact angle is commonly used to measure wettability [9]. For example, lower solid surface energy results in higher contact angles for a given liquid. A surface with a contact angle >90 °C is regarded as hydrophobic, whereas a surface with a contact angle <90 °C is considered hydrophilic [10]. In addition, waterproofing materials could be classified into flexible and stiff waterproof materials [11]. The flexible waterproof materials have the characteristics of flexibility and high durability, meanwhile, they are weak at adhesion. Rigid cementitious waterproofing materials are frequently employed; however, most hard cementitious waterproof materials are surface-sealing waterproofing agents [12].

Acrylic polymer like other polymers is inexpensive, simple to process, electrically and thermally insulator, chemically resistant, and has lower strength and modulus, as well as lower usage temperature restrictions. Also, a polymer can be degraded when exposed to UV radiation and certain solvents over an extended time. Polymerization is the process of producing large molecules from little ones or linking numerous monomers (the fundamental building blocks) together to produce polymers. The distinct behavior of polymers is caused by differences in molecule structure and form, molecular size or mass, and the quantity and kind of bonding [9]. Polymer properties can be improved by loading various fillers, including metallic and nonmetallic fillers. Amongst these fillers are Ag, Au, carbon nanotubes, multi-walled carbon nanotubes, graphene, cement, composites, and other many materials. Amongst Portland cement, is hydraulic cement and it becomes hard when interacting with water. Portland cement can be produced by thermally heating lime, alumina, silica iron, and a small amount of gypsum at temperatures ranging from 1450 to 1650 °C [13]. Conventional cement-based coatings are extremely brittle and hard, as well as they do not bridge hairline fissures in the concrete surface caused by environmental changes [14].

The adhesion, abrasion resistance, thermal stability, and antibacterial properties of water-based acrylic polymer/SiO_2_–Ag nanocomposite coating were studied [15]. The incorporation of multi-walled carbon nanotubes into acrylic-based bone cement shows an improvement in their mechanical and thermal stability [16]. Mechanical properties such as Young’s modulus, flexural strength, and compressive strength under the standard curing of a polymer-cement composite fabricated by in situ polymerization within the cement matrix were evaluated [17]. The thickness of hybrid epoxy and acrylic polymer coating/nanoclay shows a significant influence on their mechanical properties and thermal stability [18]. The wettability of polymer/cement composites were investigated using the contact angle, as well as the adhesion strength and antibacterial activity [19]. The impact of clay content in acrylate latex particles on structural, water absorption, and mechanical properties were investigated [20]. Few studies have been conducted to examine the contribution of chitosan and graphene oxide in the biologically active and antimicrobial activities of acrylic bone cement [21,22,23].

Consequently, there is still a need to develop acrylic polymer composite materials for a variety of applications that are low in cost and high in efficiency. Loading or doping with sufficient materials of a critical concentration is one of the important methods that could be used to improve the materials. The effect of cement nanoparticle loading, with a ratio range of 0–8 wt%, in acrylic polymer/cement nanocomposites on structural properties are investigated in this work. As a result, the impact of structural parameters on mechanical parameters such as impact strength, as well as Hardness shore A, and average flexural stress-strain behavior are assessed. Thermal stability, glass transition, and decomposition temperatures are investigated using thermal gravimetric analysis (TGA). The contact angle of the investigated compositions is used to investigate the wettability of the nanocomposites.

## 2. Materials and Methods

### 2.1. Materials

Acrylic polymer solution (white) coating was purchased from Al Gurg Fosroc LLC, and A.G.C.C., UAE. Similarly, ordinary Portland cement (grey) with a component composition of 21.9 wt% SiO_2_, 6.9 wt% Al_2_O_3_, 3.9 wt% Fe_2_O_3_, 63 wt% CaO, 2.5 wt% MgO, and 1.7 wt% SO_3_, manufactured by Al Gurg Fosroc LLC and A.G.C.C., UAE.

### 2.2. Preparation of Samples

**Figure 1:**
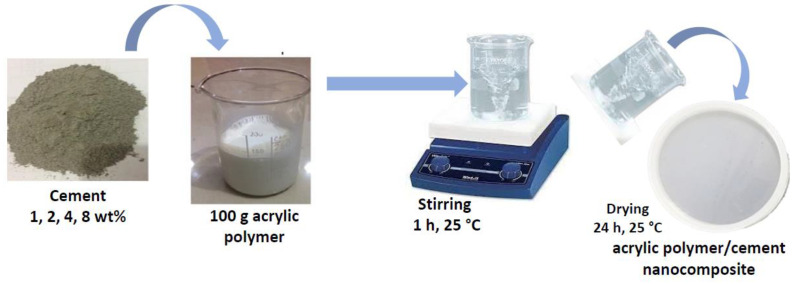
A schematic shows the procedures for preparing acrylic polymer composites.

Around 100 g of the acrylic polymer solution was prepared by the casting method at room temperature as illustrated schematically in Figure 1. To make acrylic polymer/cement nanocomposites using the casting technique, cement powder in percentages of 0, 1, 2, 4, and 8 wt% was incorporated into the polymer solution. The produced solution was then put into a glass tube on a magnetic stirrer for an hour at room temperature (25 °C). The composite was then kept at room temperature for 24 h. The casted acrylic polymer/cement nanocomposite has a diameter of 20 cm, and a thickness of 1 mm. Figure 1 depicts a schematic diagram of the above procedures used to prepare acrylic polymer solution/cement nanocomposites.

### 2.3. Characterization of Samples

Atomic force microscopy (AFM) was used to examine the surface morphology of cement and silica powder particles. This test was held at the University of Baghdad’s Department of Chemistry, College of Science, Iraq. A field emission scanning electron microscope (FE-SEM) model Mira 3 LMU (Tescan, Brno, Czech Republic) was used to examine the morphology of the acrylic polymer/cement nanocomposite.

Thermal gravimetric analysis (TGA) model Q600 Shimadzu was applied to evaluate the thermal stability of the acrylic polymer/cement nanocomposites. Around 3–5 mg of the investigated samples were heated up from 0 to 1000 °C at a heating rate of 10 °C/min.

The sessile drop method used a contact angle system to determine the contact angle of acrylic polymer/cement nanocomposites. The water contact angle was recorded on a smooth sample surface at 45–60 s intervals. For each measurement, the contact angle was monitored at specific time intervals and stored as snapshots.

Surface Roughness Analyzer, Large LCD shows either roughness parameter Ra or Rz at the press of a button, combines with the set cut-off length, and measures surface textures to traceable standards. External calibration of roughness measurements is possible through a dedicated CAL button, which simplifies modifications and provides an audible indicator when each measurement is complete.

Durometer shore-A hardness of the cured material tested as penetration. The measurements were carried out using ASTM HT-6510 A, a kind of A durometer. The indenter is pushed down into the fabric with a constant force, resulting in a scratch. This examination was conducted in the packaging center, at the Ministry of Industry and Minerals.

The impact strength was estimated by determining the minimum height from which the 20 mm diameter impactor would fall, causing mechanical damage to the coating. The test consisted of an impact with a 2 kg impactor at a height of 1 m. This examination was conducted in the Packaging Center, Ministry of Industry and Minerals.

Smooth specimens were used for the tensile test. Tensile tests on intronLaryee were carried out at a fixed crosshead speed of 5 mm/min. ASTM D638 (Shimadzu–Japan) was used to prepare the samples [24]. The machine stander on the computerized universal testing machine was 1–100 kN.

## 3. Results and Discussions

### 3.1. Structural Properteis

**Figure 2:**
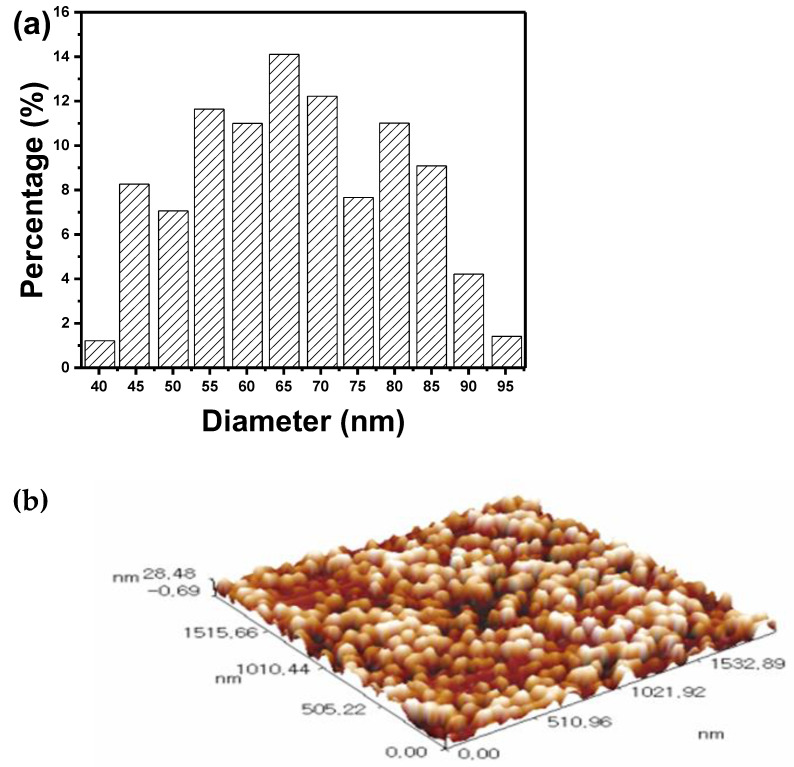
(**a**) Granularity cumulation distribution chart, and (**b**) AFM analysis images of cement particles used in the work.

The granularity cumulation distribution chart and AFM analysis images of cement particles are shown in Figure 2. The AFM reveals that the cement nanoparticles are sized uniformly. The average particle size for cement nanoparticles was estimated to be 65.25 nm. The granularity cumulation distribution chart depicts a gaussian distribution for particle size around the average value, as is typical for most AFM analyses. For cement nanoparticles, the smallest and largest particle sizes observed are 40 and 95 nm, respectively.

**Figure 3:**
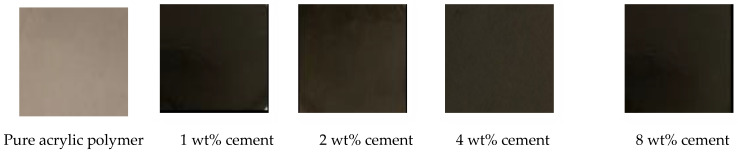
Optical images of pure acrylic polymer and acrylic polymer/cement nanocomposites.

The optical images of pure acrylic polymer and acrylic polymer/cement nanocomposites are shown in Figure 3. The color of pure acrylic polymer is light brown, and this color changes to dark brown as the cement ratio increases.

**Figure 4:**
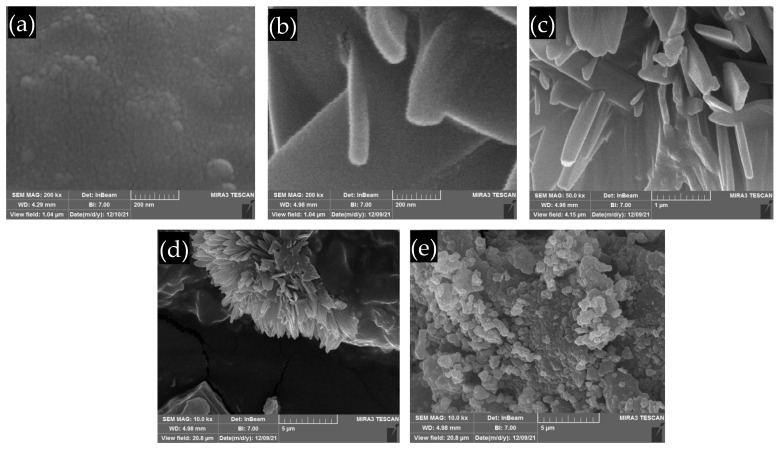
FE-SEM images of (**a**) pure acrylic polymer, and acrylic polymer/cement nanocomposites contains (**b**) 1 wt% (**c**) 2 wt% (**d**) 4 wt% (**e**) 8 wt% cement.

FE-SEM images were used to assess the agglomeration of cement nanoparticles in an acrylic polymer coating matrix. The top-view FE-SEM images for pure acrylic polymer and acrylic polymer/cement nanocomposites are shown in Figure 4. The FE-SEM image of pure acrylic polymer in Figure 4a shows a smooth surface with no discernible morphology. Depending on the cement ratio in the nanocomposites, the presence of cement in the acrylic polymer/cement nanocomposites results in the formation of various morphologies such as plates, flowers, and spherical morphologies. As shown in Figure 4b,c, plate-like morphology was observed for acrylic polymer/cement nanocomposites containing 1 wt% or 2 wt% cement. In Figure 4d, flower-like morphology was observed for acrylic polymer/cement nanocomposites containing 4 wt% cement. The spherical morphology of acrylic polymer/cement nanocomposites containing 8 wt% cement is shown in Figure 4e. The acrylic polymer coating modified by 8 wt% cement nanoparticles had more agglomeration of nanoparticles than other acrylic polymer nanocomposites. The presence of nanofiller agglomerations within the acrylic polymer microstructure was evident; the extent of these agglomerations was determined by the cement concentration. Similar results are obtained when SiO_2_-Ag and multi-walled carbon nanotube fillers are used [15,16].

### 3.2. Surface Roughness and Wettability

**Figure 5:**
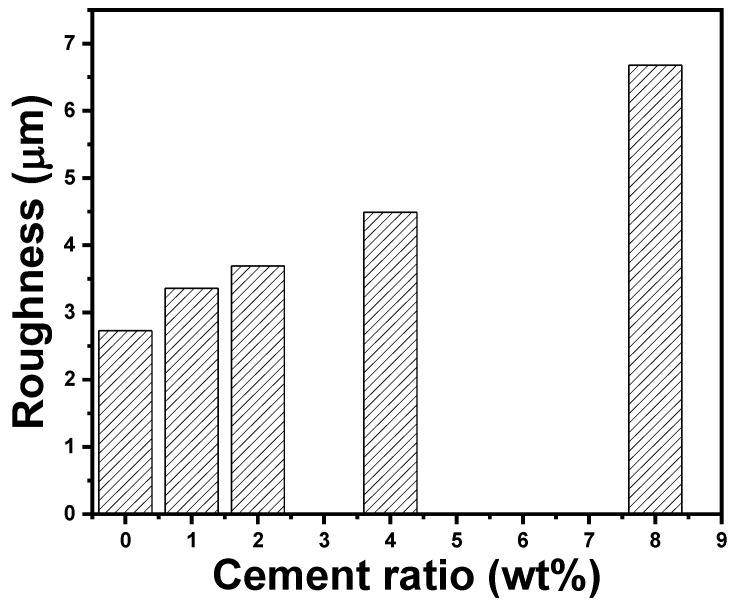
Roughness surface values of acrylic polymer/cement nanocomposites.

Figure 5 shows that introducing cement enhanced the surface roughness (Ra) of the acrylic polymer/cement nanocomposites. The roughness of the acrylic polymer/cement nanocomposites increased steadily as the cement ratio in the nanocomposites increased. The roughness of pure acrylic polymer is 2.73 μm, while it increases to 3.36, 3.69, 4.49, and 6.68 μm as the cement ratio is increased to 1, 2, 4, and 8 wt%, respectively. The surface profile of the acrylic polymer samples was noticeably smoother than that of the composites containing cement nanoparticles. The average Ra value of the acrylic polymer was 2.73 μm, but the Ra value of the cement-based composites was only 6.68 μm, indicating that adding cement resulted in a considerable increase in surface roughness. When compared to acrylic polymer, the surface roughness of nanocomposites rose 144.6% at 8 wt% cement.

**Figure 6:**
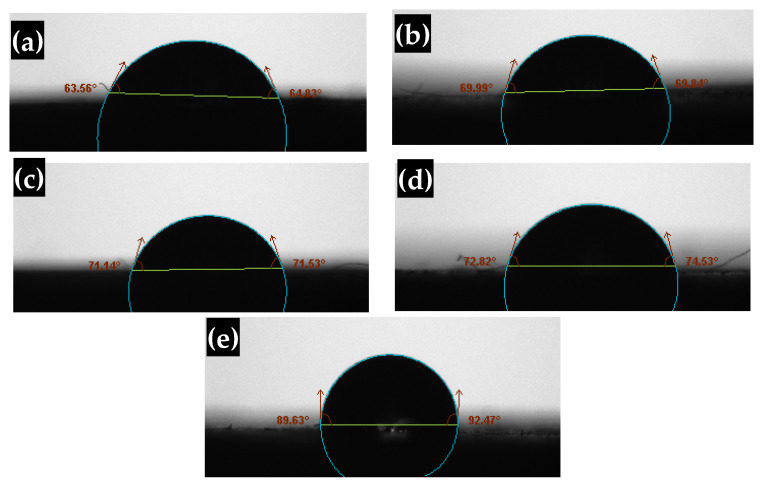
Photographs of water contact angle on the surface of (**a**) pure acrylic polymer, and acrylic polymer/cement nanocomposites contains (**b**) 1 wt% (**c**) 2 wt% (**d**) 4 wt% (**e**) 8 wt% cement.

The surface interacts with or adheres to droplets of water defining if it is a hydrophilic or hydrophobic surface. The water drop will bead up with a contact angle greater than 90° on a hydrophobic surface, whereas the drop will bead up with a contact angle less than 90° on a hydrophilic surface. The water-wetting behavior of pure acrylic polymer and acrylic polymer/cement nanocomposites has been studied. We focused on the impact of filler concentration on the wetting surface. Figure 6 depicts a polymer covering cement nanocomposites in cement nanofiller concentration with water. The contact angle was determined at the right and left sides and then the average was considered and summarized in Table 1. This observation reveals the appeal of a nonpolar surface to a polar liquid. The presence of cement nanoparticles in the acrylic polymer surface improves its hydrophobic properties. By the addition of 8 wt% cement to the nanocomposites, the contact angle of acrylic polymer coating with water was increased from 64.19° to 91.05°, resulting in a non-wetting surface. An increase in the cement filler results in a rise in the contact angle. For example, the contact angle increased to 127° as the cement ratio was increased to 25 wt% [19]. The surface-treated nanofillers, as well as the organic tail of the surface treatment, interact effectively with the polymer chain, increasing system homogeneity. The inherent features of cement nanofillers and their homogeneous dispersion respond to nanocomposites’ non-wettable surfaces [25].

The cement filler concentration has been found to have a significant effect on the wetting surface. The addition of cement fillers increased the hydrophobic nature of the composites. The increase in the wetting surface of the acrylic polymer coating with cement nanocomposites indicates that a nonpolar surface repels a polar liquid. The addition of cement nanofillers makes the polymer surface more non-polar, i.e., hydrophobic, and increases the contact angle. The nanofillers have been surface treated, and the organic tail of the surface treatment interacts well with the polymer chain, increasing system homogeneity. The intrinsic properties of nanofillers and their effective dispersion provide an answer to nanocomposites’ non-wettable surfaces.

### 3.3. Mechanical Properties

**Figure 7:**
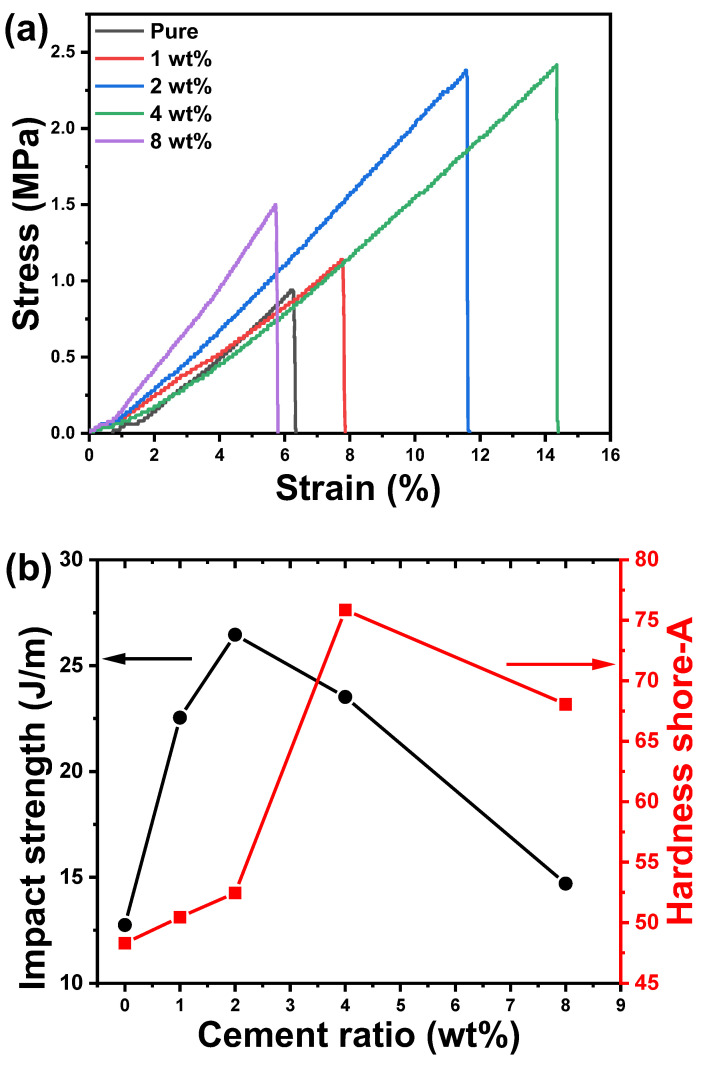
(**a**) The average flexural stress-strain curves, (**b**) impact strength (left-hand side), and hardness shore-A (right-hand side) for acrylic polymer/cement nanocomposites.

Figure 7a shows the average flexural stress-strain curves for the acrylic polymer/cement nanocomposites. It was shown that increasing the amount of cement increases the tensile strength of the polymer composites by 4% compared to a pure acrylic polymer. This observation fully demonstrated the effect of nano-inorganic particles on coating material modification. Because the system was cement nanomaterials, it had stronger intermolecular force with polymer matrix due to its advantages of a large surface area and strong adhesion, reflecting a synergy between the two components and thus showing an increase in the average flexural stress and strain which agrees with Ref. [26]. As can be seen in the figure, as the cement content increased, the tensile strength of the coating increased, and as the cement content increased, more hydrates were formed, including calcium silicates hydrates (CaH_2_O_4_Si) and calcium hydroxide Ca(OH)_2_ [27]. The geometrically complex substances increased the material’s density and tensile strength. The addition of cement, on the other hand, would decrease the free stretching of the large polymer chain, resulting in less flexibility of the organic material network and less elongation at the coating’s break. The decreased tensile strength of 4 and 8 wt% cement could be attributed to poor cement dispersion, which is a common issue for cement nanofiller.

The impact strength and hardness shore-A of pure acrylic polymer and acrylic polymer/cement nanocomposites are shown in Figure 7b. Both the impact strength and hardness of shore-A increase as the cement ratio increases, and then decrease as the cement ratio increases further. The maximum impact strength value was found in acrylic polymer/cement nanocomposites containing 2 wt% cement, while the maximum hardness shore-A value was found in nanocomposites containing 4 wt% cement. By increasing the cement by a small concentration (~4 wt%), the impact strength and hardness of shore-A were increased by more than 50%. However, as the cement ratio increases, the behavior changes, which could be attributed to the agglomeration of cement nanoparticles inside the polymer composites. Acrylic polymer/cement nanocomposites were repeatedly impacted in the same location until mechanical destruction damage of the samples obtained in the impact resistance test was determined and then summarized. Meanwhile, some samples were not damaged because the addition of the weight ratio of cement nanoparticles improved the film preparation characteristics. The polymer film could be thought to have slowed the propagation of tiny cracks in cement mortar by forming an interpenetrating structure with the modified cement mortar with lower rigidity. The geometrical complex substances resulted in higher density and impact strength of the material. However, the addition of cement would also reduce the free stretching of the large polymer chain, leading to lower flexibility of the organic matter network. The improvements in mechanical properties could be attributed to the cement arresting/retarding crack propagation through the polymer by providing a bridging effect in the wake of the crack, normal to the direction of crack growth [16].

As a result of the particles’ good interaction and dispersion, there is better adhesion between the cement and the polymer coating matrix. The results show an increase in the hardness of the polymer coating at different cement content as a result of excellent interaction between cement particles and polymer matrix at 4 wt% and good particle dispersion, increasing the surface area of the filler. There was a significant improvement, especially at high cement content (8 wt%). Several factors can influence the mechanical properties of polymer composites, including polymer matrix properties, filler particle size and morphology, particle loading and distribution, and interfacial adhesion between filler particles and matrix. Increasing the cement weight ratio to 8 wt% reduces hardness values because of particle aggregation and other higher cement content [26]. With an increase in cement particles, there is a greater chance that the particles will be aggregated and form a separate phase in the polymer matrix, reducing the surface area of the particles and lowering the adhesion between the polymer matrix and cement particles.

### 3.4. Thermal Stability

**Figure 8:**
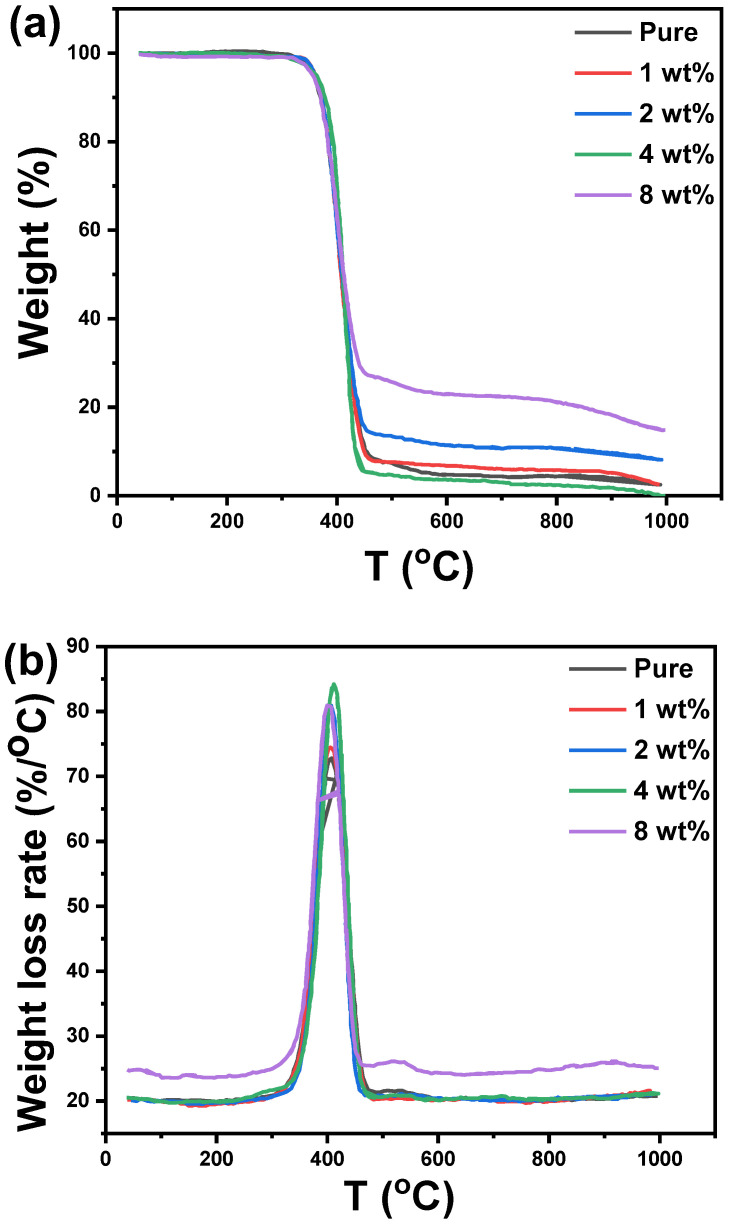
(**a**) TGA and (**b**) DTG curves for pure acrylic polymer and acrylic polymer/cement nanocomposites.

TGA can provide data on chemical processes such as desolation (particularly dehydration), decomposition, and oxidation or reduction. The TGA furthermore provides information on physical phenomena, such as second-order phase transitions, such as vaporization, sublimation, absorption, and desorption. Furthermore, the TGA is used to evaluate polymer thermal stability. The thermal stability of the acrylic polymer addition with cement nanocomposite was evaluated using TGA. Figure 8a depicts the TGA curves of acrylic polymer and its composites with varying cement content. The coatings undergo initial thermal degradation with a 5% weight loss around 300–350 °C. The release of low molecular organic substances and adsorbed water could explain the weight loss. The thermal stability of these coatings is slightly higher in this initial degradation temperature range when compared to a pure acrylic polymer. All the TGA bends demonstrated a one-step corruption mechanism, indicating that the presence of cement did not significantly alter the degradation mechanism of the matrix polymers. The results show that as the cement content increases, the acrylic polymer/cement nanocomposites become highly cross-linked, and the thermal weight loss of the composites decreases. Derivative thermogravimetry (DTG) for pure acrylic polymer and acrylic polymer/cement nanocomposites is shown in Figure 8b. The maximum peaks for polymers nanocomposites containing 0, 1, 2, 4, and 8 wt% cement, according to the DTG curves, occur at temperatures of 407, 405, 405, 411, and 399 °C, respectively. The thermal stability of acrylic polymer/cement nanocomposites improved as the degradation process was shifted to higher temperatures.

The weight loss at 1000 °C, glass transition temperature ($T_{g}$), and decomposition temperature ($T_{d}$) of various acrylic polymer/cement nanocomposites evaluated for TGA traces were summarized in Table 2. The minimum and maximum weight loss values were observed for acrylic polymer/cement nanocomposites containing 8 wt% and 4 wt%, respectively. The average glass transition temperature is around 406 ± 5 °C. As the cement filling ratio increased to 8 wt%, the decomposition temperature was raised from 450 °C for the pure acrylic polymer to 525 °C.

## 4. Conclusions

Acrylic polymer/cement nanocomposites were made by the casting method. The incorporation of cement nanoparticles with weight concentrations of (0, 1, 2, 4, and 8 wt%) to acrylic polymer achieves high-quality specifications, good adhesion, and an improvement of the mechanical and thermal properties. The average flexural stress and sample roughness improved to maximum values in the acrylic polymer/cement composites as the cement amount increased to 4 wt% and 8 wt%, respectively. The amount of cement nanofiller used influenced the mechanical properties of the resulting acrylic/cement polymer nanocomposite. The maximum values of the impact strength and hardness of shore A were observed at cement ratios of 2 wt% and 4 wt%, respectively. The decrease in mechanical properties at higher concentrations of cement nanofiller could be attributed to cement nanoparticle agglomeration and poor distribution in the polymer matrix. As the weight ratios of cement additives increase, the contact angle increases, indicating that the prepared coating is not hydrophilic and does not allow water permeability through it. The results show that the acrylic polymer/cement nanocomposites with an 8 wt% cement ratio are the best nanocomposites for high-efficiency waterproofing.

## Figures and Tables

**Table 1:** Contact angle of pure acrylic polymer and acrylic polymer/cement nanocomposites.

Cement Content (wt%)	Contact Angle		Average Contact Angle (θ)
	Left Angle (θ_1_)	Right Angle (θ_2_)	
0	63.56	64.83	64.19
1	69.99	69.84	69.91
2	71.14	71.53	71.33
4	72.82	74.53	73.67
8	89.63	92.47	91.05

**Table 2:** Total weight loss at 1000 °C, glass transition temperature ($T_{g}$), and decomposition temperature ($T_{d}$) for acrylic polymer/cement nanocomposites.

Cement Ratio (wt%)	Total Weight Loss (wt%)	*T_g_* (°C)	*T_d_* (°C)
0	97.75	406.6	450
1	97.68	410.08	460
2	91.92	409.48	450
4	100	411.85	455
8	84.93	401.52	525

## Data Availability

Not applicable.
